# Machine learning-based prediction of cognitive outcomes in de novo Parkinson’s disease

**DOI:** 10.1038/s41531-022-00409-5

**Published:** 2022-11-07

**Authors:** Joshua Harvey, Rick A. Reijnders, Rachel Cavill, Annelien Duits, Sebastian Köhler, Lars Eijssen, Bart P. F. Rutten, Gemma Shireby, Ali Torkamani, Byron Creese, Albert F. G. Leentjens, Katie Lunnon, Ehsan Pishva

**Affiliations:** 1 Medical School, Faculty of Health and Life Sciences, University of Exeter, Exeter, UK; 2 Department of Psychiatry and Neuropsychology, School for Mental Health and Neuroscience (MHeNs), Maastricht University, Maastricht, The Netherlands; 3 Department of Advanced Computing Sciences, FSE, Maastricht University, Maastricht, The Netherlands; 4 Department of Medical Psychology, Radboud University Medical Center, Nijmegen, The Netherlands; 5 Department of Bioinformatics—BiGCaT, School of Nutrition and Translational Research in Metabolism (NUTRIM), Maastricht University, Maastricht, The Netherlands; 6 Department of Integrative Structural and Computational Biology, Scripps Research, La Jolla, CA 92037 USA

**Keywords:** Parkinson's disease, Predictive markers

## Abstract

Cognitive impairment is a debilitating symptom in Parkinson’s disease (PD). We aimed to establish an accurate multivariate machine learning (ML) model to predict cognitive outcome in newly diagnosed PD cases from the Parkinson’s Progression Markers Initiative (PPMI). Annual cognitive assessments over an 8-year time span were used to define two cognitive outcomes of (i) cognitive impairment, and (ii) dementia conversion. Selected baseline variables were organized into three subsets of clinical, biofluid and genetic/epigenetic measures and tested using four different ML algorithms. Irrespective of the ML algorithm used, the models consisting of the clinical variables performed best and showed better prediction of cognitive impairment outcome over dementia conversion. We observed a marginal improvement in the prediction performance when clinical, biofluid, and epigenetic/genetic variables were all included in one model. Several cerebrospinal fluid measures and an epigenetic marker showed high predictive weighting in multiple models when included alongside clinical variables.

## Introduction

Cognitive impairment and dementia are highly common and debilitating non-motor symptoms in Parkinson’s Disease (PD). Cognitive impairment in PD carries distinct diagnostic challenges, a higher burden of care, worse functioning, and a lower quality of life^1^. Cross-sectional population studies show that ~30% of cases with PD have dementia, with 20–25% of patients presenting with mild cognitive impairment (MCI)^2^ as early as diagnosis^3^. Longitudinal studies report an average of 50% of PD patients develop dementia within 10 years^4,5^. Despite this high prevalence, however, significant cognitive impairment in the early stage of the disease is often underdiagnosed in most clinical settings^6^, in part due to the complex and multi-domain nature of cognitive dysfunction in PD^7^. Several demographic and clinical measures have been shown to be predictive in PD-cognitive impairment, including age, visual hallucinations, REM sleep disorder, and severity of parkinsonism, in particular non-tremor symptoms^1^. Moreover, considerable research interest has focused on identifying objective biomarkers, including structural and functional imaging, biofluid measures, and genetic risk^8–10^.

A major challenge for predicting cognitive outcome in PD is the high levels of heterogeneity implicit within the condition, with high interindividual variation in clinical presentation and progression^11^. A potential solution for addressing such challenges is utilizing algorithms that combine multiple measures for individual-level cognitive outcome prediction^12–14^. Employing multivariate panels of data, however, comes with limitations implicit in the complexity of multi-modal data. Compared to classical statistical methodology, learning-based methods benefit from being able to process high-dimensional and complex data, finding both linear and nonlinear associations and extracting meaningful variables of interest^15,16^. Therefore, a growing area of research opts to utilize machine learning (ML) approaches both to identify data-driven subtypes of disease^17,18^ and to predict disease progression^19–21^ including future cognitive outcomes^14,22^.

In the present study, we assessed longitudinal records of cognitive diagnoses in the Parkinson’s Progression Markers Initiative (PPMI)^23^, a well-characterized cohort of early PD patients and used multiple ML methods to predict cognitive outcome using baseline variables. We assessed prediction of two outcome measures over an 8-year time period: (i) development of cognitive impairment (MCI or dementia) and (ii) development of dementia. Variables were split into three subsets, including clinical measures, biofluid (CSF, serum) assays and variables of genetic/epigenetic markers in blood. These variables were tested separately and in combination, to assess the performance of ML methods.

For prediction, we applied four different machine learning algorithms (Random Forest [RF], ElasticNet, Support Vector Machines [SVM] and Conditional inference forest [Cforest]) and assessed the performance of each to determine if different learning approaches show better overall predictive accuracy. Applying multiple outcome measures, different subsets of predicting variables and ML algorithms, we aimed to test which showed the best overall predictive performance, establish powerful multivariate predictive models, and highlight important predictive variables included in these models.

## Results

### Prediction of cognitive outcomes

**Fig. 1:**
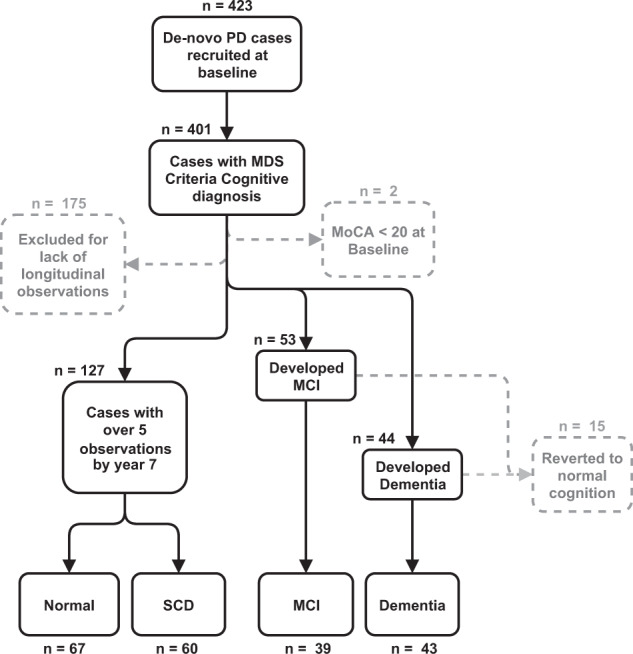
Flow diagram of case subsetting criteria. Samples retained in each stage are shown as black lines between boxes, samples excluded shown as dotted gray lines and boxes. Case numbers for each selection stage are shown overlaid on each plot. Final subset groups (Normal, SCD, MCI, and Dementia) are shown at the bottom of the flow diagram. MDS Movement Disorder Society, MoCA Montreal Cognitive Assessment, MCI Mild Cognitive Impairment, SCD Subjective Cognitive Decline.

**Fig. 2:**
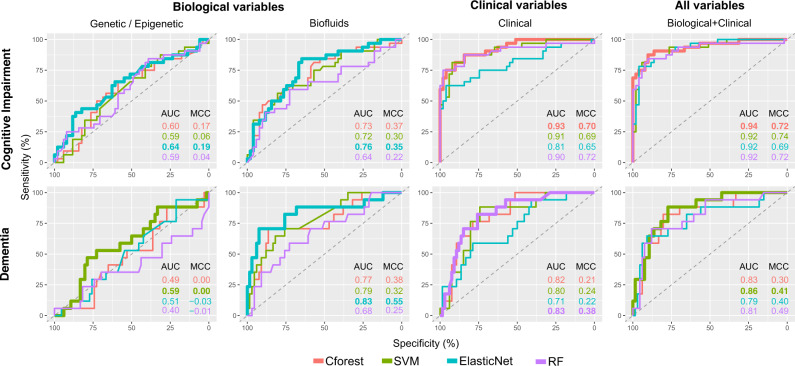
Receiver operating characteristic plots for predicting cognitive impairment and dementia using selected clinical, genetic/epigenetic, and biofluid variables. ROC curves displayed in grid with rows as cognitive outcome and columns as variable subset. Colored by ML algorithm with the highest AUC for each outcome and variable set displayed as a thicker line. AUC and MCC metrics displayed as text for each plot. ROC receiver operating characteristic AUC area under the curve, MCC Matthews Correlation Coefficient, ML machine learning, SVM Support Vector Machines, Cforest Conditional Inference Random Forest, RF Random Forest.

Using records of cognitive diagnosis over an 8-year time period (Fig. 1), we subset two cognitive outcomes. The first outcome tested development of overall cognitive impairment, including a group showing solely normal or subjective cognitive decline (SCD) (*n* = 127) and another with development of MCI and Dementia (*n* = 82). The second outcome tested dementia development; comparing a dementia conversion group (*n* = 43) to a set of combined normal, SCD and MCI cases (*n* = 166) (Fig. 1). Four ML algorithms were used for prediction using baseline variables, with each evaluated based on metrics of overall accuracy. Descriptive statistical summaries of each cognitive outcome group tested are shown in Table 1. Baseline variables were binned into individual subsets of genetic/epigenetic (47 variables), biofluid (12 variables), and clinical (64 variables) measures (Summarized in Supplementary Table 1) and tested individually and collectively. An overview of individual ML algorithm accuracy for each variable subset and outcome are summarized in Fig. 2 and Table 2.

**Table 1:** Summary statistics of demographic and selected clinical measures.

	Cognitive impairment		Dementia conversion	
Variable name	Cognitively intact	Cognitively impaired	Non-dementia	Dementia
Age at baseline	60.0 (9.14)	66.4 (8.68)***	61.6 (9.58)	66.4 (8.03)
Sex (female/male)	45/82	18/66*	53/114	10/34
Years of education	15.9 (2.76)	15.6 (3.17)	16.0 (2.69)	15.0 (3.64)
Duration of disease since diagnosis (months)	6.21 (6.43)	7.47 (7.14)	6.90 (6.66)	6.01 (7.05)**
Age at PD diagnosis	59.5 (9.13)	65.8 (8.75)***	61.0 (9.55)	65.9 (8.13)**
Hoehn & Yahr Stage (0/1/2/3)	0/67/59/1	0/33/51/0	0/82/84/1	0/18/26/0
MDS-UPDRS Part III Score (OFF)	18.8 (7.8)	22.7 (8.9)*	19.7 (8.23)	22.7 (8.92)**
Benton Judgement of Line Orientation Score	13.4 (1.64)	12.0 (2.47)**	13.1 (1.96)	11.8 (2.41)***
Geriatric Depression Scale Score	1.91 (2.23)	2.90 (2.45)**	2.22 (2.42)	2.61 (2.18)
HVLT immediate/total recall	26.7 (4.40)	20.9 (5.03)***	25.3 (5.29)	20.8 (4.67)***
HVLT delayed recall	9.48 (1.90)	6.74 (2.83)***	8.79 (2.53)	6.86 (2.69)***
HVLT delayed recognition	11.5 (0.789)	10.6 (1.510)***	11.2 (1.170)	10.8 (1.360)**
HVLT false alarms	0.976 (1.02)	1.520 (1.38)**	1.050 (1.12)	1.730 (1.39)**
HVLT discrimination recognition	10.40 (1.59)	8.69 (2.84)***	10.00 (2.1)	8.59 (2.81)***
HVLT retention	0.913 (0.132)	0.786 (0.278)**	0.881 (0.192)	0.789 (0.267)**
Letter Number Sequencing Score	11.20 (2.56)	9.04 (2.59)***	10.80 (2.68)	8.80 (2.66)***
Semantic Fluency Total Score	51.9 (10.60)	41.3 (9.12)***	49.6 (11.10)	40.3 (8.96)***
STAI Total Score	61.6 (15.5)	70.2 (18.1) **	63.7 (16.1)	70.0 (19.7)**
Symbol Digit Modalities Score	44.6 (7.43)	34.2 (9.64)***	42.7 (8.49)	31.8 (9.63)***
MOCA Score (adjusted for education)	27.9 (1.74)	26.0 (2.82)***	27.3 (2.23)	26.4 (2.93)**

For each outcome, summary values of mean (standard deviation) for continuous measurements or proportions for categorical variables. Significance values reported as the results of a Mann–Whitney U test for continuous and a Chi-2 test for categorical variables (*P < 0.5, **P < 1.0 E-3, ***P < 1.0 E-5).

*PD* Parkinson’s disease, *MDS-UPDRS* Movement Disorder Society Unified Parkinson’s Disease Rating Scale, *HVLT* Hopkins Verbal Learning Test, *STAI* State Trait Anxiety Inventory.

**Table 2:** Summary of predictive accuracy for individual ML methods.

Outcome	Variable set	Algorithm	Number of variables	MCC	AUC	TP	TN	FP	FN	Accuracy	Balanced accuracy	Sensitivity	Specificity	PPV	NPV	AUC CI (95%)	
																Lower bound	Upper bound
Cognitive impairment	Clinical + biological	**Cforest**	**28**	**0.721**	**0.938**	**23**	**49**	**2**	**9**	**0.867**	**0.840**	**0.719**	**0.961**	**0.920**	**0.845**	**0.882**	**0.993**
		*SVM*	*19*	*0.744*	*0.925*	*26*	*47*	*4*	*6*	*0.880*	*0.867*	*0.812*	*0.922*	*0.867*	*0.887*	*0.866*	*0.985*
		ElasticNet	24	0.692	0.925	25	46	5	7	0.855	0.842	0.781	0.902	0.833	0.868	0.868	0.981
		RF	11	0.719	0.917	26	46	5	6	0.867	0.857	0.812	0.902	0.839	0.885	0.846	0.989
	Clinical	**Cforest**	**11**	**0.702**	**0.930**	**21**	**50**	**1**	**11**	**0.855**	**0.818**	**0.656**	**0.980**	**0.955**	**0.820**	**0.876**	**0.984**
		SVM	7	0.693	0.911	23	48	3	9	0.855	0.830	0.719	0.941	0.885	0.842	0.845	0.976
		ElasticNet	20	0.646	0.806	20	49	2	12	0.831	0.793	0.625	0.961	0.909	0.803	0.700	0.913
		*RF*	*8*	*0.718*	*0.905*	*24*	*48*	*3*	*8*	*0.867*	*0.846*	*0.750*	*0.941*	*0.889*	*0.857*	*0.825*	*0.985*
	Biofluid	*Cforest*	*5*	*0.366*	*0.731*	*12*	*47*	*4*	*20*	*0.711*	*0.649*	*0.375*	*0.922*	*0.750*	*0.701*	*0.616*	*0.845*
		SVM	4	0.304	0.718	12	45	6	20	0.687	0.629	0.375	0.882	0.667	0.692	0.604	0.833
		**ElasticNet**	**4**	**0.347**	**0.756**	**20**	**37**	**14**	**12**	**0.687**	**0.675**	**0.625**	**0.725**	**0.588**	**0.755**	**0.649**	**0.863**
		RF	4	0.219	0.636	21	29	22	11	0.602	0.613	0.656	0.569	0.488	0.725	0.506	0.766
	Genetic/ epigenetic	Cforest	16	0.168	0.597	14	37	14	18	0.614	0.582	0.438	0.725	0.500	0.673	0.469	0.724
		SVM	7	0.064	0.594	8	41	10	24	0.590	0.527	0.250	0.804	0.444	0.631	0.469	0.718
		**ElasticNet**	**8**	**0.190**	**0.645**	**14**	**38**	**13**	**18**	**0.627**	**0.592**	**0.438**	**0.745**	**0.519**	**0.679**	**0.520**	**0.771**
		RF	6	0.045	0.594	14	31	20	18	0.542	0.523	0.438	0.608	0.412	0.633	0.467	0.721
Dementia	Clinical + biological	Cforest	14	0.303	0.826	5	62	4	12	0.807	0.617	0.294	0.939	0.556	0.838	0.716	0.936
		**SVM**	**10**	**0.409**	**0.862**	**8**	**60**	**6**	**9**	**0.819**	**0.690**	**0.471**	**0.909**	**0.571**	**0.870**	**0.772**	**0.951**
		ElasticNet	23	0.401	0.791	11	53	13	6	0.771	0.725	0.647	0.803	0.458	0.898	0.653	0.928
		*RF*	*9*	*0.489*	*0.809*	*9*	*61*	*5*	*8*	*0.843*	*0.727*	*0.529*	*0.924*	*0.643*	*0.884*	*0.688*	*0.930*
	Clinical	Cforest	10	0.207	0.824	4	61	5	13	0.783	0.580	0.235	0.924	0.444	0.824	0.727	0.922
		SVM	8	0.239	0.801	4	62	4	13	0.795	0.587	0.235	0.939	0.500	0.827	0.691	0.911
		ElasticNet	27	0.216	0.709	5	59	7	12	0.771	0.594	0.294	0.894	0.417	0.831	0.572	0.845
		**RF**	**8**	**0.382**	**0.828**	**8**	**59**	**7**	**9**	**0.807**	**0.683**	**0.471**	**0.894**	**0.533**	**0.868**	**0.725**	**0.930**
	Biofluid	Cforest	7	0.382	0.767	8	59	7	9	0.807	0.683	0.471	0.894	0.533	0.868	0.629	0.906
		SVM	6	0.320	0.786	4	64	2	13	0.819	0.603	0.235	0.970	0.667	0.831	0.667	0.905
		**ElasticNet**	**5**	**0.546**	**0.835**	**8**	**64**	**2**	**9**	**0.867**	**0.721**	**0.471**	**0.970**	**0.800**	**0.877**	**0.704**	**0.966**
		RF	6	0.250	0.676	6	58	8	11	0.771	0.616	0.353	0.879	0.429	0.841	0.529	0.823
	Genetic/ epigenetic	*Cforest*	*9*	*0.000*	*0.492*	*0*	*66*	*0*	*17*	*0.795*	*0.500*	*0.000*	*1.000*	*NA*	*0.795*	*0.343*	*0.642*
		**SVM**	**16**	**0.000**	**0.594**	**0**	**66**	**0**	**17**	**0.795**	**0.500**	**0.000**	**1.000**	**NA**	**0.795**	**0.439**	**0.750**
		ElasticNet	5	−0.026	0.510	1	61	5	16	0.747	0.492	0.059	0.924	0.167	0.792	0.362	0.658
		RF	5	−0.006	0.403	3	54	12	14	0.687	0.497	0.176	0.818	0.200	0.794	0.228	0.578

Summary table of metrics evaluating accuracy of ML predictions. Lower and higher confidence intervals (CI) show 95% CI for AUC. Bold lines show best AUC measures per variable set tested, italic lines show best MCC measures per variable set tested.

*TP* true positive (impaired/dementia), *TN* true negative (cognitively intact/non-dementia) (cognitively intact/non-dementia), *FP* false positive, *FN* false negative, *PPV* positive predictive value, *NPV* negative predictive value.

Comparing both outcomes, prediction of cognitive impairment outcome showed better predictive accuracy than dementia conversion, reflected by higher area under the receiver operating characteristic curve (AUC) and Matthews Correlation Coefficient (MCC) metrics for all variable subsets. The one exception to this was biofluid variables, which when evaluating solely on AUC, appeared to show better prediction of dementia conversion than cognitive impairment. However, reviewing the prediction of dementia using biofluid variables shows poor overall prediction of true dementia converters when investigating MCC (Cforest = 0.38, SVM = 0.32, ElasticNet = 0.55, RF = 0.25) and sensitivity metrics (Table 2).

Overall, across both outcomes and variable sets, the best prediction was achieved for the cognitive impairment outcome using a combination of biological and clinical variables, reflected by high value balance for AUC and MCC (Table 2). This represented a marginal improvement over prediction of the cognitive impairment outcome using the clinical variable subset alone. Combining biological and clinical variable types improved sensitivity over the clinical models, represented by a higher number of true cognitive impairment predictions (Table 2).

The genetic/epigenetic variables alone showed minimal predictive accuracy irrespective of cognitive outcome and ML algorithm tested, with near-random prediction, with AUC measures between 0.40 and 0.65 and MCC below 0.19 (Fig. 2, Table 2).

### Predictive variables for cognitive impairment outcome

**Fig. 3:**
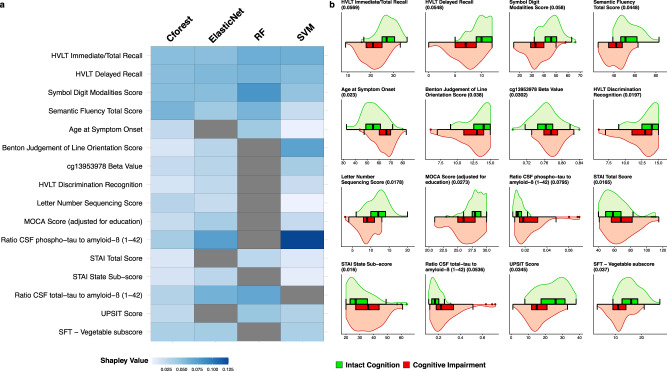
Variable importance in predicting cognitive impairment outcome. Variables included across three or more ML models for prediction of the cognitive impairment outcome using combined clinical and biological variables. **a** A heatmap of global Shapley importance. Darker blue reflects higher Shapley value and more important variables in the model. Variables not included in a particular model are shown in gray. **b** Dual violin and box plots of raw values of each variable between groups. Average global Shapley value importance for each variable is shown in brackets next to each variable name. Boxes represent median, Q1 and Q3 of the interquartile range (IQR) and whiskers display 1.5× IQR below and above Q1 and Q3, respectively. HVLT Hopkins Verbal Learning Test, MOCA Montreal Cognitive Assessment, CSF cerebrospinal fluid, STAI State-Trait Anxiety Inventory, UPSIT University of Pennsylvania Smell Identification Test, SFT semantic fluency test, ML machine learning.

Given the best overall prediction was achieved using a combination of biological and clinical variables for the cognitive impairment outcome, predicting development of both MCI and dementia, we further investigated individual variable contribution using Shapley values. Shapley values can be interpreted as the additive relative importance of a particular variable to a model’s prediction (Methods). Variables included by at least three ML algorithms are shown in Fig. 3. Cognitive tests were heavily represented in overlapping models, with Hopkins Verbal Learning Test-Revised (HVLT-R) Immediate/Total Recall and Delayed Recall scores, Symbol Digit Modalities (SDM) and Semantic Fluency Test (SFT) being included in all four ML methods and Benton Judgment of Line Orientation (BJLO), HVLT-R Discrimination Score, Montreal Cognitive Assessment (MoCA), and SFT—Vegetable subscore being included in at least three (Fig. 3a).

Noncognitive clinical measures included in multiple models were age of symptom onset, State Trait Anxiety Inventory (STAI) scores (total and state subscore) and the University of Pennsylvania Smell Identification test (UPSIT) for olfactory impairment. In these combined models, three biological variables showed consistently high contribution across multiple models including CSF Ratios of phospho-tau to amyloid-β (1–42) and total-tau to amyloid-β (1–42), respectively, as well as blood DNA methylation at cg13953978 (Fig. 3a). Differences in overlapping variables are shown in Fig. 3b, highlighting the direction of effect for each variable between cognitively intact and impaired groups.

Looking at correlation between top predictive variables included across multiple models, we found that eight show collinearity (Pearson’s Correlation > 0.7), including HVLT Immediate and Delayed Recall, Semantic Fluency Total Score and SFT—Vegetable subscore, STAI total and state subscores and CSF Ratios of phospho-tau to amyloid-β (1–42) and total-tau to amyloid-β (1–42). By contrast eight variables: SDM, age of symptom onset, BJLO, methylation at cg13953978, HVLT discrimination score, LNS, MOCA, and UPSIT all show a higher degree of independence (all Pearson’s Correlations < 0.6).

Genetic variables were conspicuous in their absence from overlapping contributing variables, but were present in certain models, for example, *GBA* nonsynonymous mutations were included for both Cforest and ElasticNet. Summarized Shapley value contribution across all tested algorithms are shown in Supplementary Figs. 1–4. As a graphical representation of prediction in our best performing model (Cforest), Supplementary Fig. 5 displays a surrogated decision tree, built by aggregating the best performing decision trees within the forest, containing a mix of biological and clinical variables. It is worth noting that this representation does not contain all variables included in the entire decision forest.

### The effect of cognitive tests in predictive accuracy

**Fig. 4:**
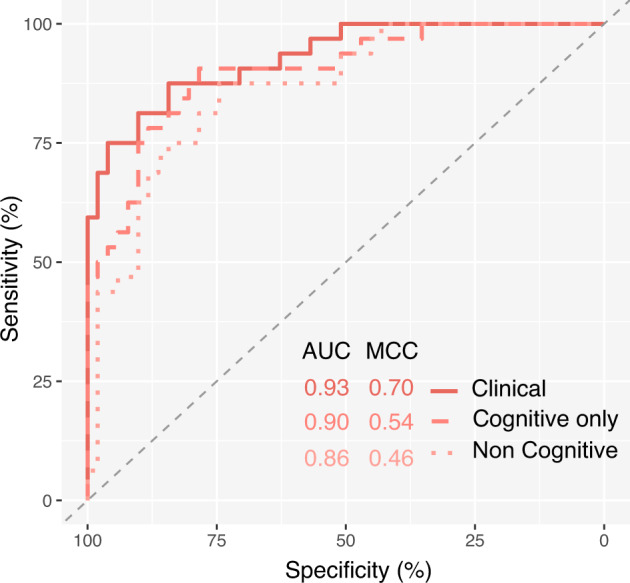
Sensitivity analysis of cognitive variables. ROC showing prediction of the cognitive impairment outcome using Cforest applied on clinical subsets. Noncognitive variables: dotted line, cognitive variables: dashed line, all clinical variables: solid line. Summary of AUC and MCC metrics for each subset shown in plot text. AUC area under the curve, MCC Matthews Correlation Coefficient, Cforest Conditional Inference Random Forest, ROC receiver operating characteristic.

As we observed a large proportion of the top predictive variables were cognitive tests (9 out of 16, Fig. 3a), we tested the sensitivity of predictions made without the use of cognitive variables. As Cforest models performed best on the clinical subset, we chose to explore the sensitivity of predictions with and without cognitive variables using this algorithm. Clinical variables were subset to cognitive only and noncognitive variables as annotated in Supplementary Table 1. As shown in Fig. 4, we found that cognitive variables only (AUC = 0.90, MCC = 0.54) performed better than noncognitive variables (AUC = 0.86, MCC = 0.46). The combination of the two variable subsets into an overall clinical model showed a marginal increase in AUC (0.90–0.93) but a larger increase in sensitivity reflected by increased MCC from 0.54 to 0.70.

### Stratification of PD- MCI from PD-dementia

Given that MCI represents an intermediate stage between normal cognition and dementia, we next tested if ML methods could accurately distinguish 43 PD-dementia from 39 PD-MCI, without records of further progression to dementia with the same length of follow-up time (Supplementary Fig. 9). Across variable subsets, we observed best individual performance from clinical variables with increased performance in combination with biological variables. However, models lacked overall accuracy in their predictions (AUC 0.69–0.75), in particular with lower MCC values (0.177–0.470), reflecting a low specificity (0.4–0.6) of dementia prediction. All these results together indicate that in this context, the generated ML models lack accuracy to resolve dementia from MCI over the timescale tested.

## Discussion

In the present study, we tested the prediction of two cognitive outcome measures in newly diagnosed PD subjects within 8 years, using multiple variable subsets and ML algorithms. The generated models were assessed for metrics of prediction accuracy and the importance of contributing variables. We found that combining both biological and clinical variables produced best performing models, with a marginal improvement in predictive performance compared to models using clinical variables alone. We interpret this as evidence of synergistic contribution of multivariate data types, producing the most accurate predictions. Of variable subsets, the most accurate and balanced prediction was achieved when testing for cognitive impairment (MCI and dementia combined) using clinical data, giving the highest AUC, MCC metrics and balance of sensitivity and specificity. When evaluated individually, nonclinical measures (biofluids and genetic/epigenetic) showed poor predictive performance, regardless of outcome tested and ML algorithm used.

Comparing outcomes, prediction of combined cognitive impairment, merging cases developing either MCI or dementia, consistently outperformed dementia conversion alone, which we interpret as being driven by poor differentiation of MCI individuals when predicting dementia conversion. Indeed, models tested to stratify MCI from dementia cases performed poorly with low specificity of predictions. MCI is a well-established risk factor for future dementia development^4^, and previous studies show higher dropout within PPMI is associated with worse cognitive performance^24^. Given this, the overall progression profile of MCI and dementia, as subsets within this study, might not differ substantially, with MCI patients potentially converting to dementia in unobserved events. This further supports the use of a combined cognitive impairment group, with best prediction being observed for this outcome.

Unsurprisingly a high number of contributing variables included cognitive assessments, indicating that there was already a level of cognitive changes present at baseline. This highlights a potential limitation in the inclusion of these variables, as these cognitive assessments are highly associated with the outcome of interest we aimed to predict. However, these measures reflect an assessment time 1–7 years before a clinically diagnosed conversion to either MCI or dementia. Sensitivity analysis of the effect of cognitive variables in prediction confirmed that cognitive variables had a large contributory effect to predictions although increased sensitivity was observed with the inclusion of noncognitive clinical variables. Top contributing noncognitive variables included age at onset of PD, anxiety, and olfactory impairment. Older age of PD onset, which we observe within the cognitive decline group, is a well-established and validated risk factor for PD-cognitive decline^4^. Olfactory impairment has been increasingly associated with cognitive impairment in PD^25–28^. Although anxiety is less associated as a predictive variable for cognition within PD^29^, it has been associated as a predictor of worse cognitive prognosis in general population studies^30^.

Within combined models utilizing both biological and clinical variables, ratios of CSF protein measures of total-tau, phospho-tau and amyloid-β (1–42), had a high contributory effect across multiple ML algorithms. Additionally, one measure of blood DNA methylation, cg13953978, was included in multiple combined models. This locus has been previously associated with multiple neurodegenerative diseases and, of note, we observe the same direction of effect between cognitively impaired and preserved individuals in this study and previously reported findings^31^.

Several studies have aimed at creating an accurate model to predict cognitive outcome in PD using the PPMI cohort^13,22,32^. Compared to previous studies, in the current study, we have included a larger range of biological variables including polygenic scores for multiple related traits and epigenetic measures. We used MDS criteria for defining cognitive performance at each follow-up as a substitute for the commonly used MoCA. Additionally, we included a long follow-up period and excluded reverters from the modeling.

To improve the accuracy and generalizability of our models compared to other models reported previously, we employed a multi-objective model optimization procedure using three criteria (AUC, MCC, and number of variables). Although AUC is commonly used for model interpretation, it is insensitive to class imbalance. Therefore, to prevent inaccurate prediction assessment, we included MCC, as this metric can evaluate accuracy while considering class balance. This, along with recursive feature elimination (RFE)^33,34^ and k-fold cross-validation, further avoided the risk of overfitting and addressed the high number of variables included in this dataset. We applied multiple ML algorithms, to cover a range of different learning strategies, standardly applying RFE and multi-objective optimization for each.

A potential limitation of this study is the curatorial nature in which cognitive groups were subset and the relatively small sample size available. We justify the methods for cognitive group subsetting as we aimed to represent individuals with clinically relevant diagnoses confirmed by multiple observations over time. However, due to data missingness and attrition within PPMI, there are a number of de novo cases enrolled at baseline which were not tested within our models.

A potential caveat of this study is its broader applicability to samples outside of PPMI. Replication efforts in additional cohorts are hampered by the unique nature of PPMI as a cohort, both in how thoroughly assessed these individuals are, the early de novo stage at which they were enrolled and the longitudinal observations present, in particular in the MDS-cognitive diagnosis measure used as an outcome here. To our knowledge, a viable cohort covering these domains is not available at current.

PPMI’s de novo stage has important implications for the broader applicability when comparing to prediction models of cognitive progression in later disease stages. In Phongpreecha et al.’s 2020 study^14^, using cases from the Pacific Udall Centre (PUC) Cohort, they tested multitask models for prediction of future yearly incidence of MCI and dementia diagnosis. They report highest accuracy for prediction of dementia and retained normal cognition, with lowest accuracy for MCI prediction, largely consistent with our findings in the PPMI cohort. Furthermore, they highlight cognitive measures as the most important variables in their model in line with our findings following RFE. However, they report higher AUC measures for their dementia conversion predictions than we observed here. This may be attributable to the different distributions of the disease stage of the PUC PD patients compared to the newly diagnosed PPMI patients.

Salmanpour and colleagues^35^ have employed machine learning in the prediction of cognitive outcomes in PPMI. Our studies differ however in the cognitive outcome tested, with the use of MDS-criteria cognitive diagnosis conversion here and using MoCA at year 4. We also explored a larger range of biological measures and restricted predictor input solely to baseline, while Salmanpour incorporated measures at year 1 in the models. Differences in methodology and outcome measure make direct study comparison difficult; however, despite the variability in methodology some interesting consistencies between the two studies are evident, in particular in the finding of baseline state-trait anxiety as a predictive measure.

A previous study by Liu et al.^13^ developed a multivariate predictor of global cognitive impairment in a large multi-cohort analysis. The predictive score reported high performance, with high positive predictive (0.87) and negative predictive value (0.92) utilizing solely age at onset, MMSE, education, motor exam score, gender, depression, and GBA mutational status. This predictive model benefits from generalizability, both as a result of the high number of samples used to validate it and in the low variable number required to achieve prediction. However, the multi-center design of the study introduces a high level of heterogeneity, both in the disease stage included and the outcome measure used to define cognitive impairment^36^, something which is highly consistent within our study here. Furthermore, due to the range of variables included in PPMI, we were able to explore a broader range of biological and clinical predictors in our present study.

Our findings of DNA methylation at cg13953978 as a predictive variable requires further replication to ensure it is not the result of an unknown cryptic stratification in this cohort. Previous association of this loci with neurodegenerative disease across multiple cohorts do however support it as a potential biomarker. Expanding the number of genetic and epigenetic variables included in future studies to a genome-wide level in cohorts designed around cognitive decline prediction is also essential to truly uncover potential predictive efficacy. However, due to the challenge of including the high number of variables implicit in multi-omics data^24,37–39^, we found this to be outside of the scope of the current study.

Although not explored in this study, incorporation of neuroimaging measures in cognitive predictive models represent an important additional data modality for future work. A number of studies have highlighted structural underpinning to PD-MCI and dementia^40,41^ and in this present study we highlight four cognitive tests, consistently incorporated across multiple ML algorithms. Taking these measures of perturbed cognitive domains as indicative of structural changes in the brain, we can interpret executive dysfunction, as measured by the semantic fluency score being evidence of associated frontal lobe atrophy^41^. Some studies have associated verbal memory, as we see measured by the HVLT, with differences in the inferior frontal gyrus^42^ and in the context of PD with functional changes associated in the anterior cingulate and orbitofrontal cortex^43^. Our finding of the attentional test assessed by SDM having predictive contribution supports studies relating attentional effects to striatal dopamine in dopamine active transporter (DAT) imaging^44^ and to microstructure changes in the anterior cingulate and frontal cortex using diffusion tensor imaging (DTI)^45^.

In summary, after evaluating multiple predictive variable types and outcomes, we established a model that accurately predicted cognitive impairment and preserved normal cognition over a follow-up 8-year time span. This prediction was largely driven by clinical measures of both known risk factors and more novel measures, but also variably included biological variables. This work supports evidence of anxiety and olfactory impairment as potential predictors of cognition in PD and highlights epigenetic measures of DNA methylation as biological predictive variables requiring further investigation.

## Methods

### Participants and cognitive assessment

All data used in this study was obtained from the PPMI^18^ database (https://ida.loni.usc.edu/). Participating PPMI sites all received approval from an ethical standards committee before study initiation and written informed consent was obtained for all individuals participating in the study. The study was registered at clinicaltrials.gov (NCT01141023). Participants were selected from the de novo PD cohort, defined by a diagnosis of the disease within 2 years and unmedicated for motor symptoms at baseline (*n* = 423). Subjects underwent yearly cognitive diagnosis in accordance with Movement Disorders Society (MDS) recommended criteria for dementia and MCI as previously reported^19–21^. In brief, a confirmed MCI diagnosis was based on an impaired performance on at least two test scores >1–2 standard deviations below a standardized mean^46^. Dementia diagnosis alongside clinical annotation required impaired performance in at least two cognitive domains coinciding with significant functional impairment resulting from cognitive state^47^.

Records of cognitive diagnoses from baseline to year 8 were sourced from PPMI following their routine application of the above criteria to create three groups of PD patients with distinct cognitive outcomes as follows (Fig. 1 and Supplementary Fig. 6):

#### PD-Dementia

Cases showing any diagnosis of dementia over an 8-year time span were annotated as the dementia conversion cases, excluding one individual that reverted to normal cognition after an annotation of dementia (*n* = 43).

#### PD-MCI

Cases with any record of MCI without any annotation of future dementia diagnosis (*n* = 39) were annotated as PD-MCI conversion cases. This group excludes a set of 14 cases that reverted to normal cognition following MCI annotation.

#### Cognitively intact (CI)

To avoid any effect of attrition and cognitive decline in unobserved events, cases defined as cognitively intact required a minimum of five records of normal or subjective cognitive decline (SCD) during recorded visits up to year 8 (*n* = 127). This excluded 175 cases showing missing values or indeterminate diagnoses.

Subsequently, we used these groups to define two separate binary outcomes for machine learning-based prediction as follows:

#### Cognitive impairment outcome

Defining conversion to cognitive impairment within an 8-year time span. This compared the CI group (*n* = 127) to an impaired group, created by combining the PD-Dementia and PD-MCI groups (*n* = 82).

#### Dementia conversion outcome

Defining conversion to dementia within an 8-year time span. This compared the PD-Dementia group (*n* = 43) to a non-dementia conversion group created by combining PD-MCI and CI groups (*n* = 166).

### Epigenomic and genomic profiling

#### Genotyping and polygenic scores calculation

Whole blood DNA genotyping was previously performed on the NeuroX SNP array by PPMI investigators using published methods^48^. Raw data from 423 individuals covering 267,607 variants was quality control (QC) assessed following published recommendations^49^. In brief, data was excluded on the following criteria: (1) variants and individuals with missingness >0.1, (2) individuals with discordant reported sex and inferred sex (X chromosome homozygosity F-value >0.8 for males, <0.2 for females), (3) variants with minor allele frequency <0.01 or >0.05, (4) variants deviating from Hardy Weinberg Equilibrium <1e-3, (5) individuals with heterozygosity rate ±3 standard deviations, (6) individuals with evidence of cryptic relatedness (pi hat >0.2). Following initial QC, autosomal data was extracted, plink files were converted to vcf format and uploaded to the Michigan Imputation Server. Imputation was conducted using Eagle2 to phase haplotypes and Minimac4 using the 1000 Genomes reference panel (phase 3, version 5). An R^2^ filter score for imputation quality was set at 0.3. Following imputation, data was downloaded, converted to plink format and quality assessed following the previous criteria. Finally, genetic principal components were generated along with reference data from the 1000 Genomes Project and non-European cases removed based on qualitative assessment of clustering of the first two principal components. Five hundred eighty-two cases passed QC (total variants post-imputation *n* = 2,287,446).

Polygenic risk scores (PRS) were calculated using summary statistics from recent genome-wide association studies (GWAS) for Alzheimer’s disease (AD)^50^, PD^51^, education attainment (EA)^52^, schizophrenia (SCZ)^53^, major depressive disorders (MDD)^54^ and coronary artery disease (CAD)^55^. For AD, the effect of the *APOE* region was excluded by removing the region chr19:45,116,911–chr19:46,318,605. For PD, the effect of the *GBA* region was excluded by removing the region chr1:154,600,000 - chr1:156,600,000. PRSice-2 software^56^ was used for polygenic risk score calculation, which automates clumping and p-value thresholding to generate a “best-fit PRS” for a target phenotype of interest. Briefly, clumping was performed to retain the most significant GWAS variants in a linkage disequilibrium (LD) block (250 kb window, r2 threshold = 0.1). The PRS model is tested over an increasing set of *p*-value threshold (5e-08 to 1), with the optimal threshold set which generates a score explaining the maximum phenotypic variance in the target phenotype of interest. Phenotype was coded as a binary factor of 0 (Control) and 1 (PD) for this analysis, with the first eight genetic principal components used as covariates^57^.

#### DNA methylation data processing

Whole blood genome-wide methylation in the PPMI cohort at baseline was profiled on Illumina EPIC Array as previously reported^58^. These included individual previously associated methylated loci as well as epigenetic age prediction variables. Raw IDAT files were downloaded from the PPMI database (https://ida.loni.usc.edu/) in April 2020 and processed using the R package *wateRmelon*^59^. For epigenetic age prediction and age acceleration analysis non-normalized beta values were uploaded to the web-based tool https://dnamage.genetics.ucla.edu, selecting the “normalize data” and advanced analysis” options. For inclusion of specific epigenetic loci, data was quality controlled and normalized following established pipelines^59^. Briefly, samples with low signal intensities or bisulphite conversion rate, mismatched reported and imputed sex or cryptic relatedness were excluded. P-filtering was applied using the ‘*pfilter’* function in the *wateRmelon* package, excluding samples with >1% of probes with a detection *P*-value > 0.05 and probes with >1% of samples with detection *P*-value > 0.05. Beta values for each probe were quantile normalized using the ‘*dasen’ function*.

### Baseline data

Baseline data for all 423 PD cases were sourced from PPMI and processed into four sets of variables (Supplementary Table 1): Clinical variables: These included demographic variables (sex, age of onset, years in education, duration of disease, family history of PD), motor symptoms (MDS-UPDRS Part 2 and 3 total scores, rigidity score, tremor dominant / postural gait instability disorder classification, Hoehn and Yahr [H&Y] scale, Modified Schwab & England Activity Daily Life [ADL] Score), psychiatric symptoms (MDS-UPDRS Part 1 subscores, Geriatric Depression Scale [GDS], Questionnaire for Impulsive-Compulsive Disorders, State Trait Anxiety Test), autonomic symptoms (SCOPA-autonomic subscores), sleep disorder (Epworth Sleepiness Scale Score [ESS], Categorical REM Sleep Behavior Disorder Questionnaire subscore, MDS-UPDRS Part 1 subscores) and olfactory symptoms measured by University of Pennsylvania Smell Identification Test (UPSIT). Assessments of cognition (Semantic Fluency Test [SFT], Symbol Digit Modalities [SDM], MDS-UPDRS Part 1 subscores, Montreal Cognitive Assessment [MoCA], Hopkins Verbal Learning Test-Revised [HVLT-R] subscores, Benton Judgment of Line Orientation [BJLO]) were also included.

#### Biofluid variables

CSF measures for amyloid-β (1–42), phospho-tau181, total-tau, and α-synuclein were included, after removing cases showing high levels of CSF hemoglobin (>200 ng/mL) as previously described^60,61^. Ratios of each measure were also included as independent predictive variables. Total serum uric acid was also included as previously described^62^.

#### Genetic and epigenetic variables

Genetic variables included individual *APOE* genotype, MAPT haplotype and the SNPs rs12411216^63^, rs356181^64^, and rs3910105^65^. *GBA* mutation status was included as a binary factor for the presence of any nonsynonymous coding mutations present within the *GBA* region. PRS for PD (*GBA* region excluded), AD (*APOE* region excluded), EA, SCZ, MDD, and CAD where also included.

After stringent quality control and normalization of the whole-genome DNA methylation data measured in baseline blood, 21 loci were selected based on previously reported differentially methylated positions associated with cognitive decline in PD^66^ or across neurodegenerative disease^31^. Epigenetic age acceleration measures from the GrimAge clock^67^, BloodAndSkin clock^68^ and the modified Hannum clock which included measures of both intrinsic epigenetic age acceleration (IEAA) and extrinsic epigenetic age acceleration (EEAA, incorporating intrinsic measures as well as blood cell proportions)^69^ were included as additional epigenetic variables.

#### Combined biological and clinical variables

This variable set collated all previously listed variables across the clinical, biofluid and epigenetic/genetic subsets into one combined total set.

Summary lists of measures used for predictive modeling are shown in Supplementary Table 1 and descriptive statistics in Table 1. All measures highlighted in this summary table were carried forward for multivariate modeling.

### Data processing

#### Imputation

Each baseline variable was evaluated for the proportion of missing observations and missing values imputed using available data for the selected variable. For ordinal and categorical variables, the mode value was chosen for imputation, for continuous variables the median value was selected. Median/mode value imputation was chosen based on simulation analysis, showing better accuracy compared to k-nearest neighbors (KNN), Multivariate Imputation via Chained Equations (MICE) and Hotdeck algorithms (Supplementary Fig. 10). The full dataset was assessed on missing values, generating a value representing the missing value fraction per variable (Supplementary Table 1). Samples containing any missing value were removed to produce a dataset with complete observations for all available variables, now called the reference dataset. Missing values were induced in the reference dataset at random, according to the proportion of missing values per variable to generate a ‘test’ dataset. The imputation methods ‘Median/mode’, ‘knn’^70^, ‘hotdeck’^71^, and ‘mice’^72^ were used to impute the missing values in the test dataset. Root mean square error (RMSE) was used to determine the error between the test and reference dataset, then summed for all variables to get an overall performance error score. This process was repeated 100 times, randomizing different values per loop to be flagged as missing, to assess the stability of the imputation. The total RMSE error (mean + sd) was displayed per variable subset to indicate which methods perform best per variable type. Additionally, the proportion of missing values was compared to the average RMSE per variable.

The total error per variable subset showed the same pattern between variable subsets (Supplementary Fig. 9). The median/mode imputation showed least average error, followed by knn, hotdeck, and mice. Evaluating the proportion of missing values compared to the average RMSE, higher proportion of missing values contributes to a higher average RMSE. Median/mode imputation was chosen to apply to the actual data, as it showed the best performance in minimizing average imputation error.

#### Stratification

Due to an imbalance in the size of selected outcome groups, stratified sampling was used to account for potential training imbalance and testing bias^73^ using the ‘*stratified*’ function from the *splitshapestack* R package (version 1.4.8). Sampling considered the proportion of outcome groups, the proportion of MCI and dementia cases as well as sex and categorical age (1: <56 years, 2: 56–65 years, 3: >65 years). A 60/40 train/test split was chosen to increase samples in the test set to give an improved evaluation of the final resulting models.

#### Data transformation

The baseline data contains three types of variables: categorical, ordinal, and continuous. To ensure each variable had a similar influence during the ML process, Z-score normalization was performed using the base R function ‘*scale’* on the continuous variables based on averages of the training set^74,75^. The parameters ‘center’ and ‘scale’ were stored per variable and used to rescale the training and testing data accordingly.

### Machine learning

#### Training and selected algorithms

The R package *caret* (version 6.0.90) was used to establish the machine learning workflow and tune the hyperparameters^76^. We used four different classifiers from three machine learning families. The selected algorithms include functions for RF (‘*rf’*) and conditional inference forest (‘*cforest’*) from the RF family, SVM with linear Kernels (‘*svmLinear’*) from the support vector machine family and ElasticNet (‘*glmnet’*) from the generalized linear model family of classifiers. RF and Cforest are information-based learning algorithms, and their behavior is determined by concepts from information theory^77^. RF algorithms are based on a majority vote of a collection of different decision trees. Cforest differs from RF as it does not select variables based on maximization of an information measure but based on a permutation test for significance^78^. SVM and ElasticNet are error-based learning algorithms, and their behavior is explained by minimizing total error during training^77^. SVM algorithms are based on generating the best possible separation between classes of interest in a hyperdimensional plane. ElasticNet is a generalized linear model with L1 and L2 regularization, able to shrink or drop coefficients to achieve a better model fit.

#### Tuning

To avoid overfitting during training, 10 repeated 10-fold cross-validation was used. During the training process, hyper-tuning was enabled with a maximum of 100 tunes to promote model accuracy. To prevent optimistically inflated results due to imbalanced datasets, we used MCC alongside AUC to evaluate model accuracy^75,79^.

#### Variable selection and model generalization

Recursive feature elimination (RFE) was applied as the variable selection algorithm. In brief, RFE iterates through generations of models using a decreasing training set, eliminating the worst contributing variable of each iteration^80^. The first model was trained using all available variables, with the resulting evaluation metrics being extracted and stored. Variable importance was recursively calculated for the generated model using the ‘*varImp’* function in *caret*. The least contributing variable was flagged to be removed in the next iteration. The updated training data was used to train a new model, and the process was repeated until one variable remained. This resulted in numerous models with decreasing number of variables.

#### Optimal model selection

To reduce generalization error, a multi-objective optimization procedure was applied by utilizing MCC, AUC and the number of variables from each model in each iteration^81^. MCC and AUC were chosen as MCC is calculated on binary classes while AUC is calculated by class probability, allowing model selection to benefit from the properties of MCC and the resolution of AUC. This ensures model generalization with higher accuracy. Moving averages of these metrics (window = 5) were calculated and the rank was determined (Supplementary Figs. 7 and 8). Calculating the mean rank of the moving averages allows a comparable scale to the variable number per each *i*th model. From this we calculated an optimal model score by adding together the number of variables to the average rank, as shown in Eq. (1). This results in an optimization curve highlighting the best performing model with the lowest number of variables. The model with the lowest score was selected as the optimal model, as this model indicates the highest accuracy, balanced prediction, and least number of variables.

1 $$\begin{array}{l}{\mathrm{Optimal}}\,{\mathrm{model}}\,{\mathrm{score}}_i =\\\\ {\mathrm{number}}\,{\mathrm{of}}\,{\mathrm{variables}}_i +\, \frac{{{\mathrm{rank}}({\mathrm{MA}}({\mathrm{MCC}}_i)) + {\mathrm{rank}}({\mathrm{MA}}({\mathrm{AUC}}_i))}}{2}\end{array}$$

#### Testing

The optimal model was used for class prediction on the test dataset, yielding several evaluation metrics (AUC, MCC, Accuracy, Sensitivity, Specificity) as well as other evaluation elements (such as confusion matrices, Receiver Operator Characteristics (ROC)-AUC curves, and individual variable difference plots).

#### Variable importance calculation

Shapley values were used to assess the importance of variables included in models following RFE. Shapley values are a concept in cooperative game theory but are interpreted in the context of ML to determine a variables contribution to prediction. Shapley values were calculated for the interpretation of individual variables included in best performing models. Using the package *iml* (version 0.10.1), a predictor object was generated, containing the model of interest and the test dataset. This predictor object was used in the calculation of the Shapley values per sample, with 10,000 Monte-Carlo-Simulations. The resulting absolute Shapley values were averaged over all samples, yielding global Shapley contribution per variable^82^.

### Reporting summary

Further information on research design is available in the Nature Research Reporting Summary linked to this article.

## Supplementary information

Supplementary Figures

Supplementary Table 1

Reporting Summary Checklist

## Data Availability

Data used in the preparation of this article were obtained from the Parkinson’s Progression Markers Initiative (PPMI) database (www.ppmi-info.org/access-dataspecimens/download-data). For up-to-date information on the study, visit ppmi-info.org.

## Code availability

All codes are available at https://github.com/Rrtk2/PPMI-ML-Cognition-PD.
