# Supplementary material for: Machine learning-based prediction of cognitive outcomes in de novo Parkinson’s disease
Source: NPJ Parkinsons Dis. 2022 Nov 7;8:150. doi: 10.1038/s41531-022-00409-5 (PMC9640625; doi:10.1038/s41531-022-00409-5)
Supplement: Supplementary file 1 — Supplementary Figures [file 41531_2022_409_MOESM1_ESM.pdf]

Supplementary Material

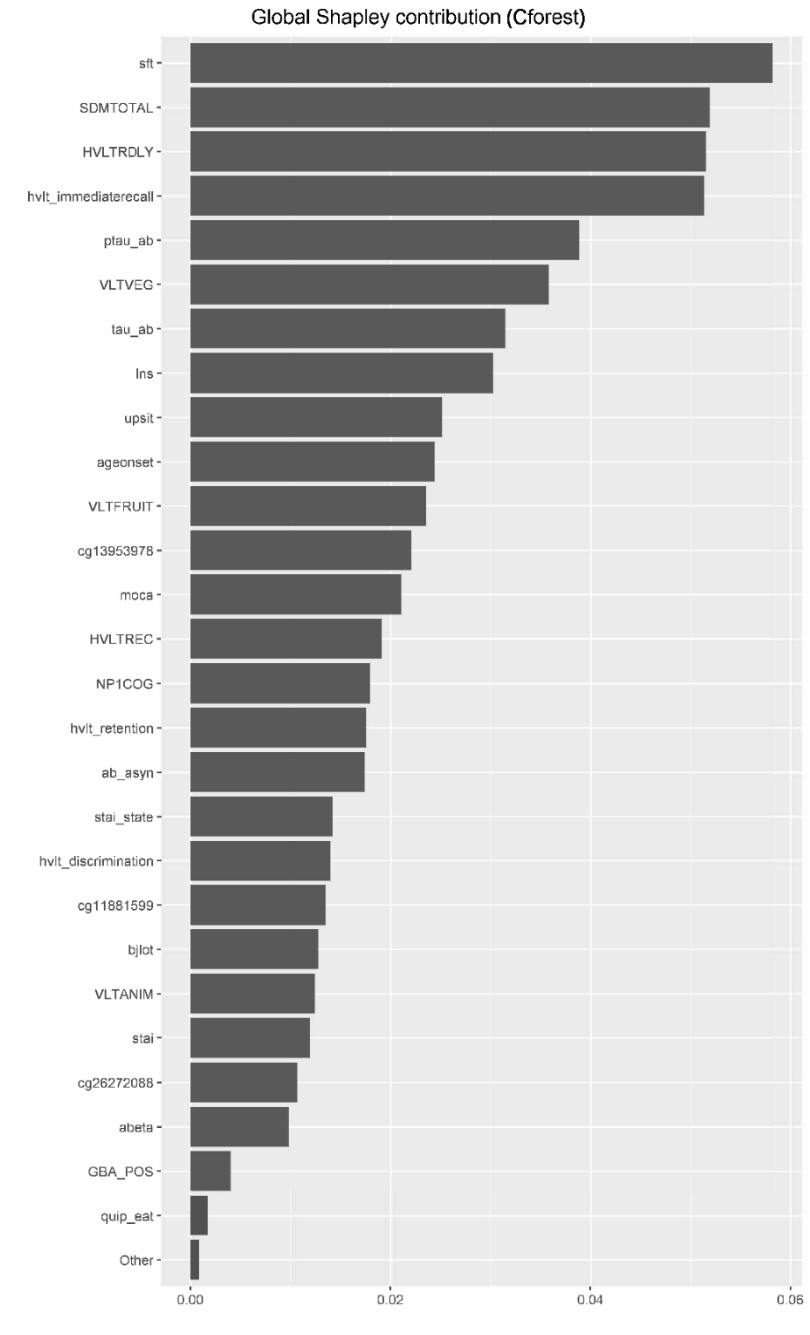

Supplementary Figure 1 Summary of variables included in Cforest ML prediction of the cognitive impairment using combined clinical and biological variable set. Raw Shapley values are shown as bar values. Variable short names are listed in Supplementary Table 1. The largest bars are at the top of the figure and represent higher Shapley value and more important variables in the model.

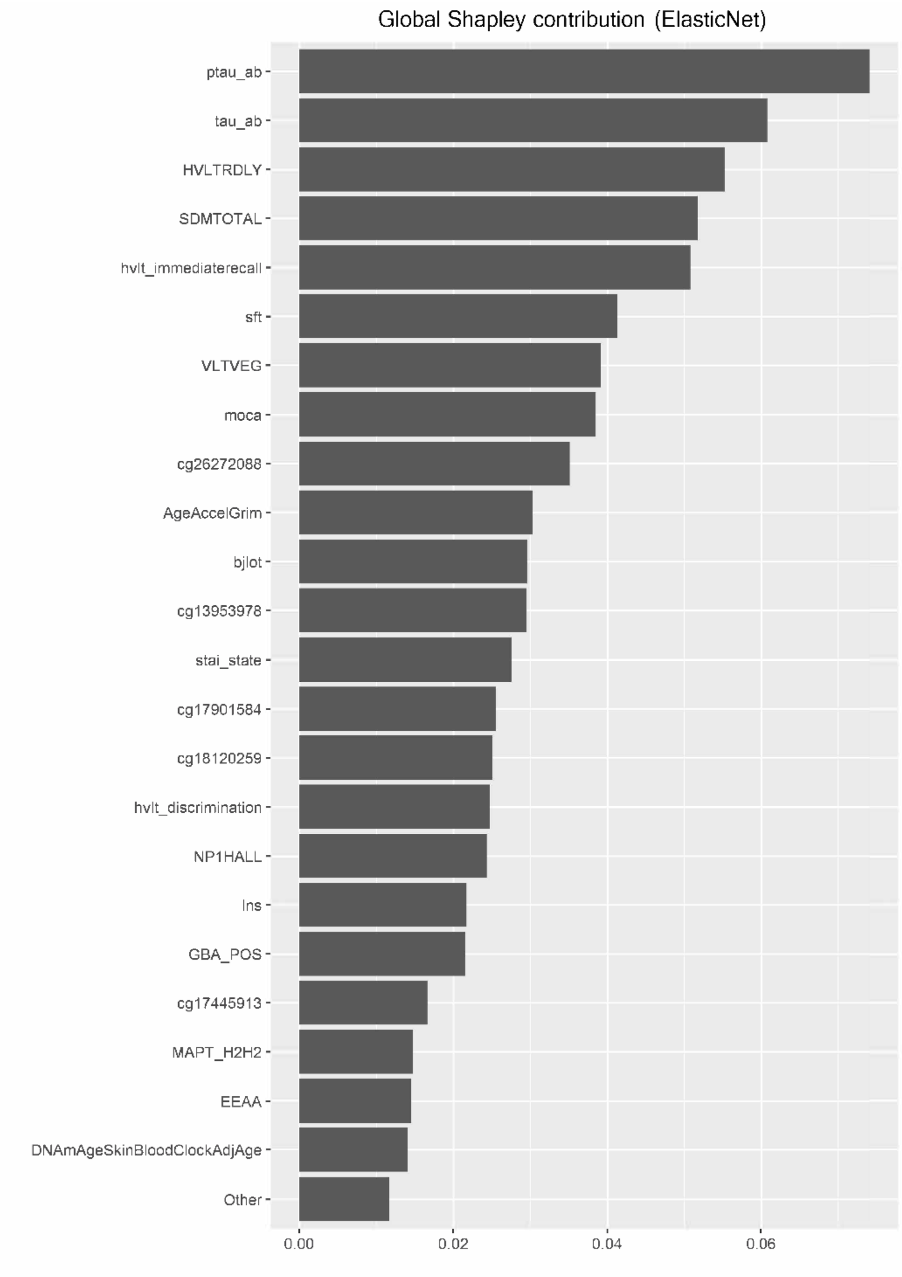

Supplementary Figure 2 Summary of variables included in ElasticNet ML prediction of the cognitive impairment using combined clinical and biological variable set. Raw Shapley values are shown as bar values. Variable short names are listed in Supplementary Table 1. The largest bars are at the top of the figure and represent higher Shapley value and more important variables in the model.

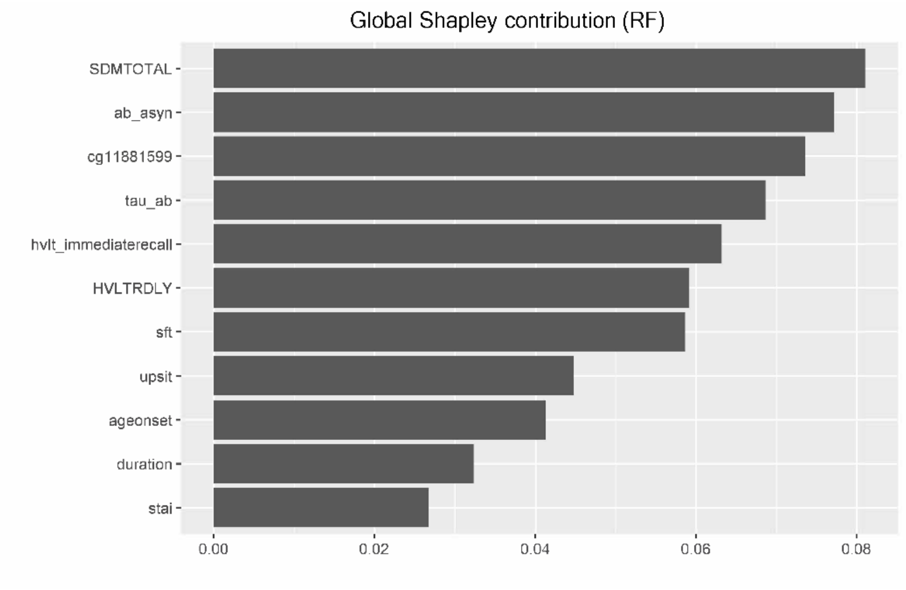

Supplementary Figure 3 Summary of variables included in RF ML prediction of the cognitive impairment using combined clinical and biological variable set. Raw Shapley values are shown as bar values. Variable short names are listed in Supplementary Table 1. The largest bars are at the top of the figure and represent higher Shapley value and more important variables in the model.

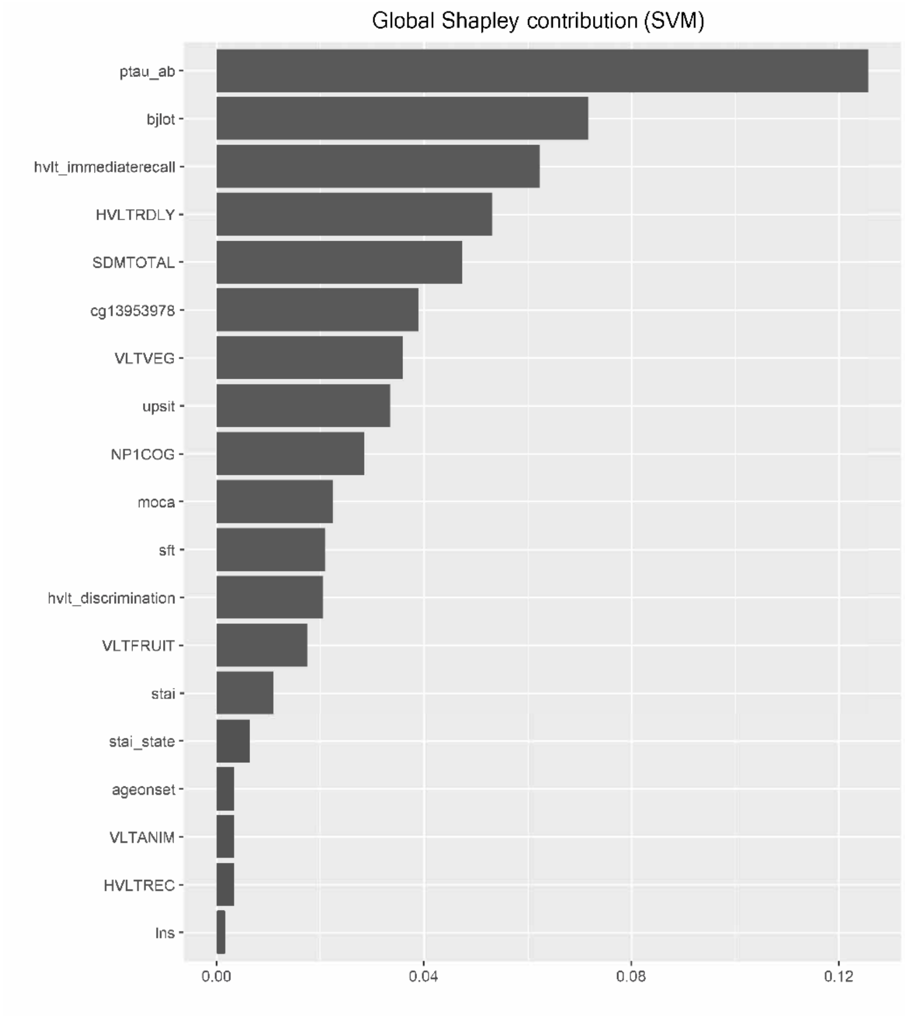

Supplementary Figure 4 Summary of variables included in SVM ML prediction of the cognitive impairment using combined clinical and biological variable set. Raw Shapley values are shown as bar values. Variable short names are listed in Supplementary Table 1. The largest bars are at the top of the figure and represent higher Shapley value and more important variables in the model.

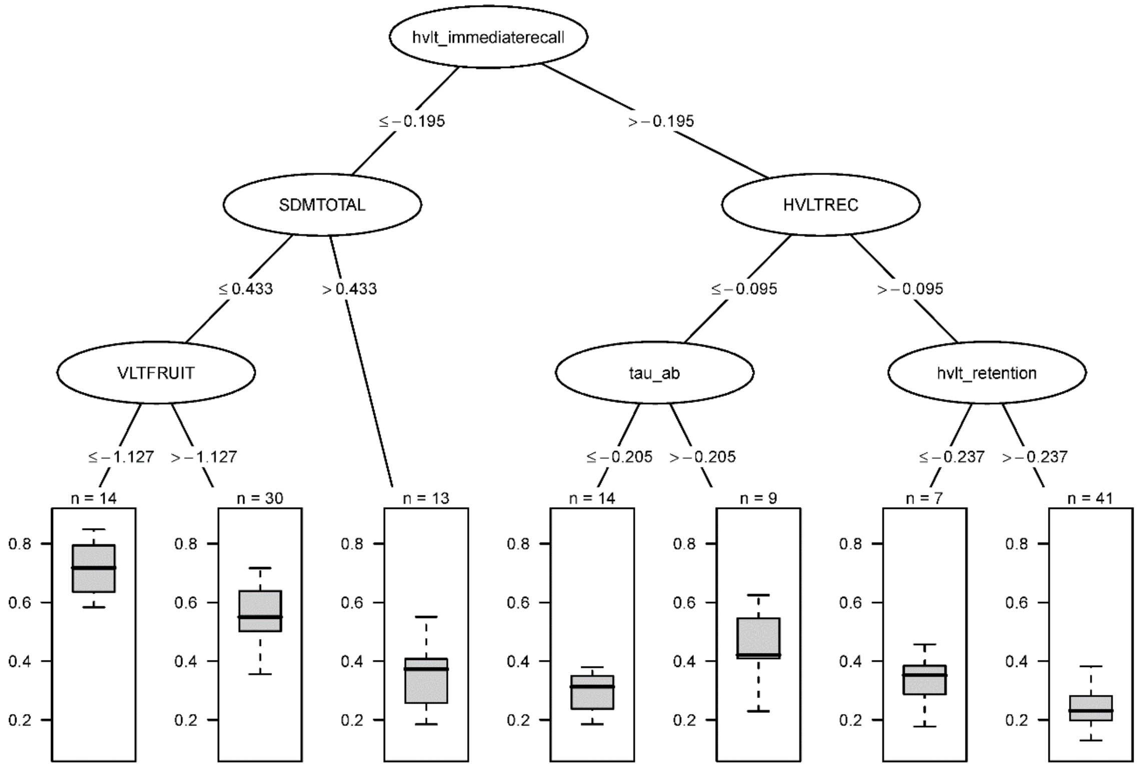

Supplementary Figure 5 Representative surrogate tree generated from full Cforest. Shown is a representative surrogated tree approximating the full forest. Nodes indicate the variables (short name as outlined in Supplementary Table 1), lines between nodes indicate the decision with corresponding criteria (Z-score normalized values, see methods), boxplots indicate probability of developing cognitive impairment. Variables are ordered on discrimination power, the first node to evaluate is hvlt_immeriaterecall, and based on the outcome the next node needs to be evaluated. Sequential decisions will result in a probability range of developing cognitive impairment shown in bottom boxplots. Number of classified training samples shown above each boxplot.

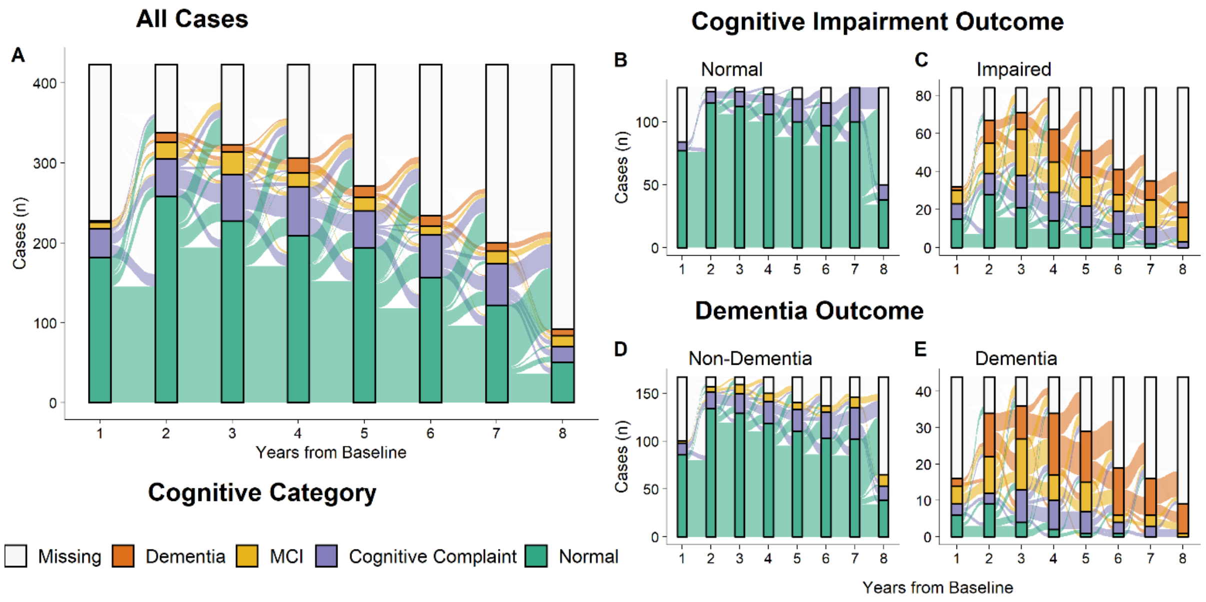

Supplementary Figure 6 Alluvial plots showing individual proportions of cognitive diagnosis for each yearly visit. The individual change in cognitive diagnosis between years represented by flow lines between nodes. **a** All enrolled de-novo PD cases in PPMI (n = 423). Subset groups for each outcome measure shown on the right-hand side. **b,c** Subset groups for the Cognitive Impairment Outcome corresponding the cognitively intact group (n = 127) and the impaired group (n = 82). **d,e** Subset groups for the Dementia Conversion outcome including the non-Dementia converting group (n = 166) and the Dementia converting group (n = 43).

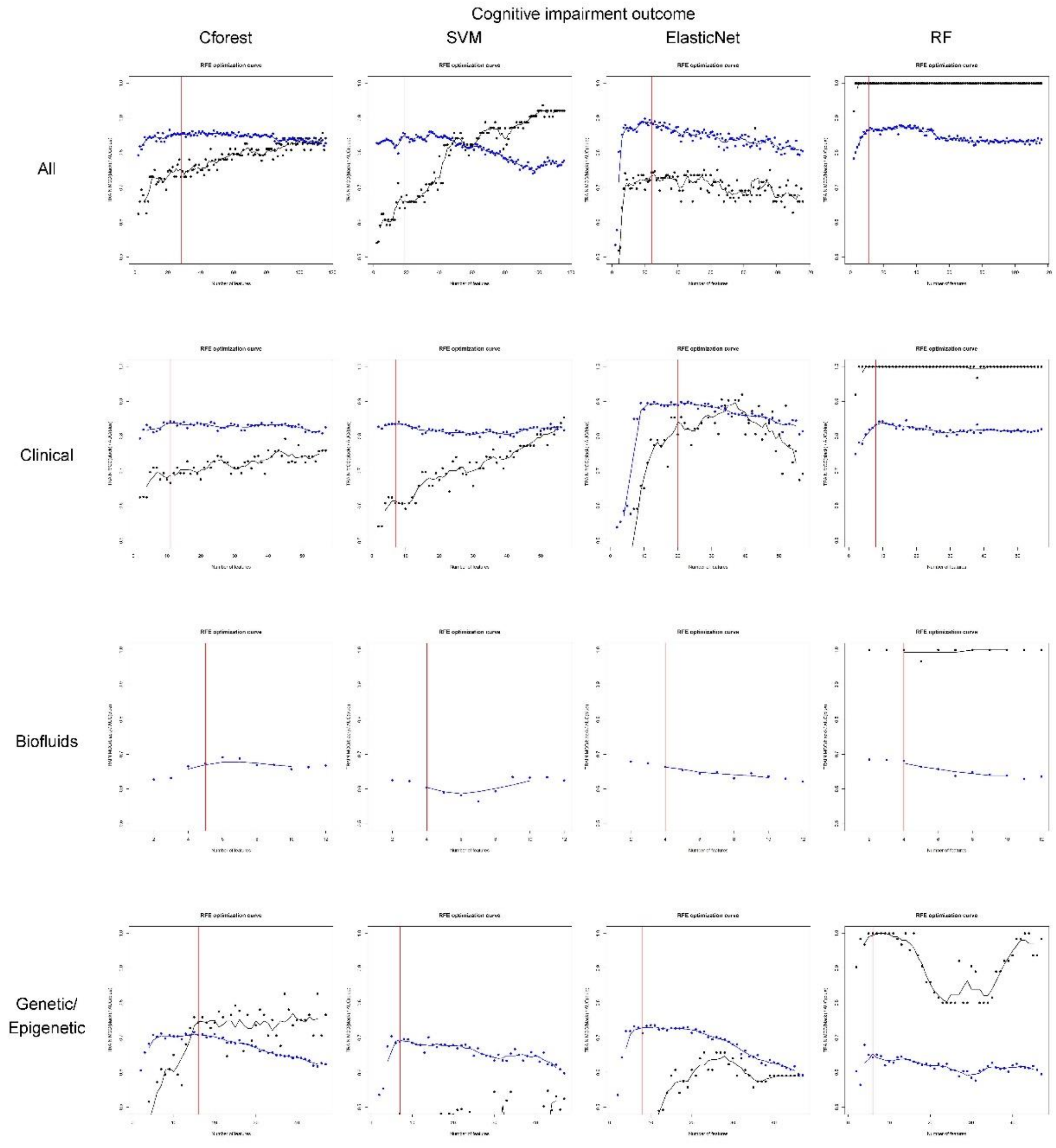

Supplementary Figure 7 Individual optimization curves for Recursive Feature Elimination for Cognitive Impairment Outcome. The number of variables added per iteration is shown along the x axis and accuracy measurements on the y axis. AUC is shown in blue and MCC in black, with lines indicating the moving average. A red line on each plot indicates the optimum model according to multi objective optimization, as detailed in the methods. Plots are organized in rows for variable subsets and columns for ML algorithms.

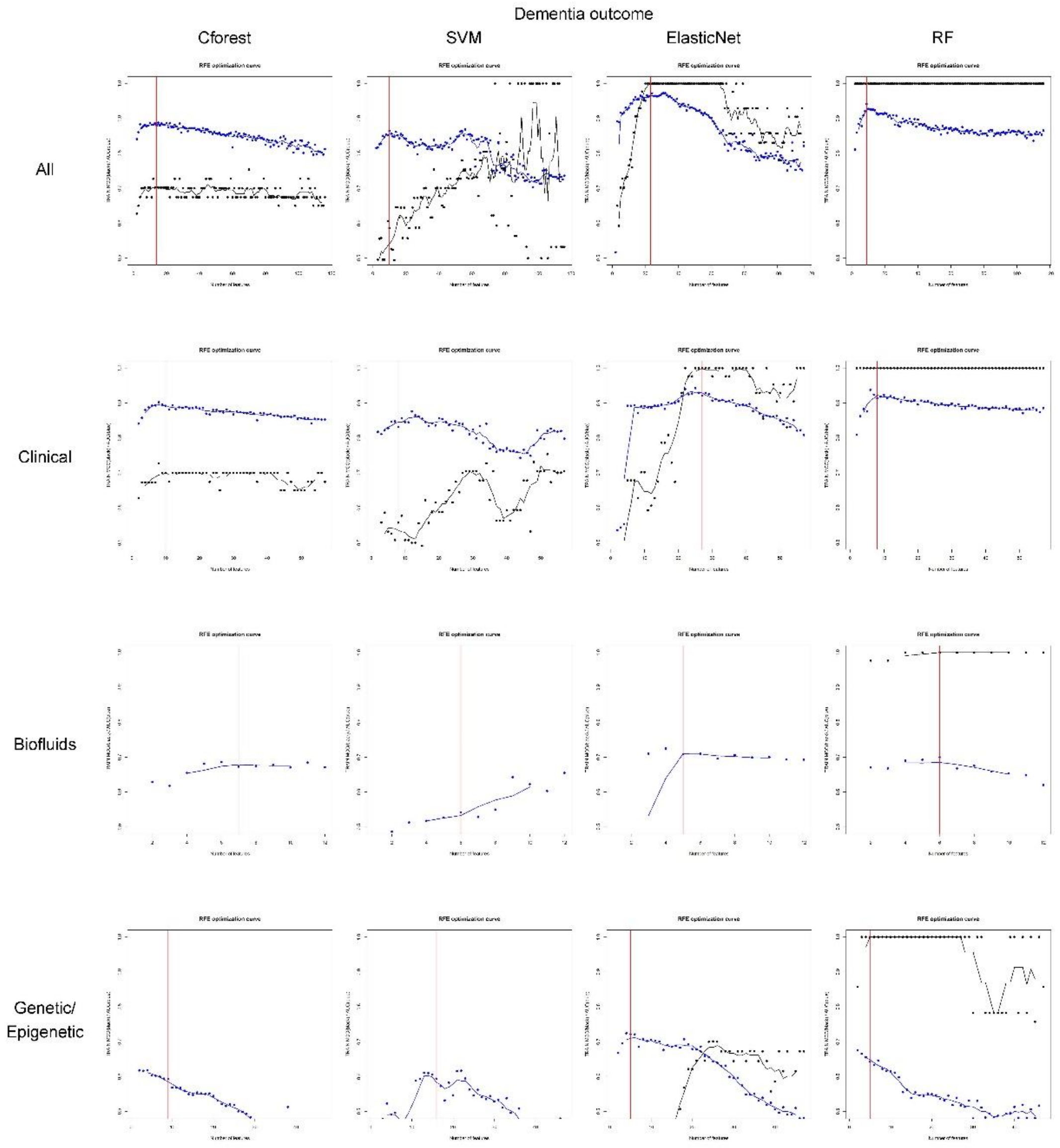

Supplementary Figure 8 Individual optimization curves for Recursive Feature Elimination for Dementia Conversion Outcome. The number of variables added per iteration is shown along the x axis and accuracy measurements on the y axis. AUC is shown in blue and MCC in black, with lines indicating the moving average. A red line on each plot indicates the optimum model according to multi objective optimization, as detailed in the methods. Plots are organized in rows for variable subsets and columns for ML algorithms.

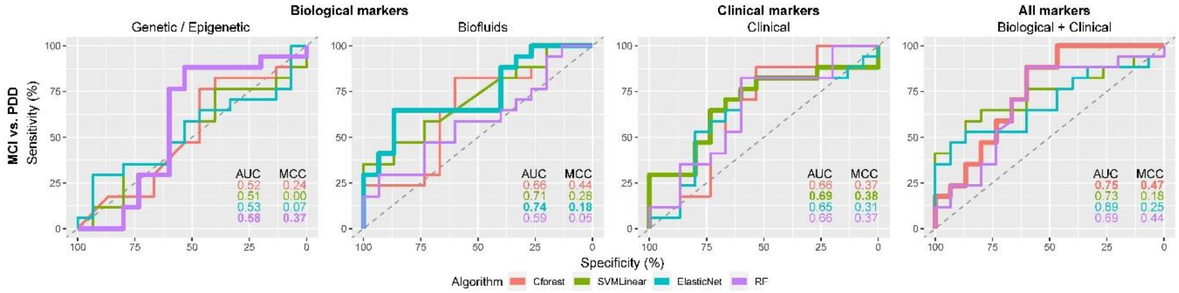

Supplementary Figure 9 Receiver operating characteristic plots for stratifying MCI cases from dementia. ROC curves displayed with columns as variable subset used to achieve prediction. Colored by ML algorithm with the highest AUC for each outcome and variable set displayed as a thicker line. AUC and MCC metrics displayed as text for each plot. ROC: Receiver Operating Characteristic AUC: Area Under the Curve, MCC: Matthews Correlation Coefficient, ML: Machine Learning, SVM: Support Vector Machines, Cforest: Conditional Inference Random Forest, RF: Random Forest

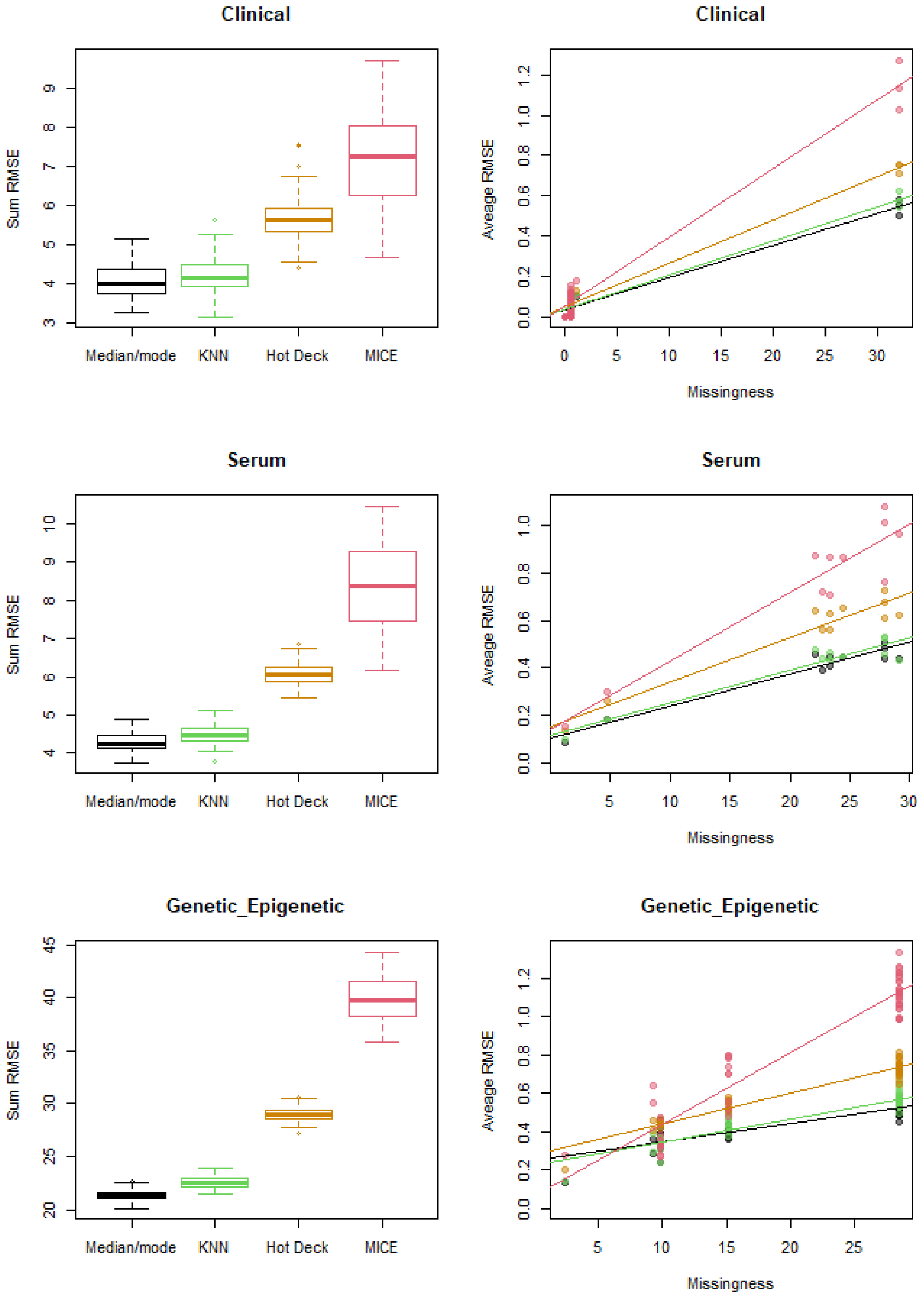

Supplementary Figure 10 Imputation error summary. Boxplots summarising average imputation root mean square error (RMSE) measured across 100 simulations. Four methods were tested, median/mode, KNN, Hot Deck and MICE. Line plots show RMSE across methods with increased induced missingness percentage per variable.
