# Supplementary material for: Machine learning-based prediction of cognitive outcomes in de novo Parkinson’s disease
Source: NPJ Parkinsons Dis. 2022 Nov 7;8:150. doi: 10.1038/s41531-022-00409-5 (PMC9640625; doi:10.1038/s41531-022-00409-5)
Supplement: Supplementary file 3 — Reporting Summary Checklist [file 41531_2022_409_MOESM3_ESM.pdf]

nature portfolio

Corresponding author(s): Dr. Ehsan Pishva
School for Mental Health and Neuroscience
Maastricht University

Last updated by author(s): Oct 3, 2022

Reporting Summary

Nature Portfolio wishes to improve the reproducibility of the work that we publish. This form provides structure for consistency and transparency in reporting. For further information on Nature Portfolio policies, see our Editorial Policies and the Editorial Policy Checklist.

Statistics

For all statistical analyses, confirm that the following items are present in the figure legend, table legend, main text, or Methods section.

n/a	Confirmed	
☐	☒	The exact sample size (*n*) for each experimental group/condition, given as a discrete number and unit of measurement
☐	☒	A statement on whether measurements were taken from distinct samples or whether the same sample was measured repeatedly
☐	☒	The statistical test(s) used AND whether they are one- or two-sided<br>*Only common tests should be described solely by name; describe more complex techniques in the Methods section.*
☐	☒	A description of all covariates tested
☐	☒	A description of any assumptions or corrections, such as tests of normality and adjustment for multiple comparisons
☐	☒	A full description of the statistical parameters including central tendency (e.g. means) or other basic estimates (e.g. regression coefficient) AND variation (e.g. standard deviation) or associated estimates of uncertainty (e.g. confidence intervals)
☐	☒	For null hypothesis testing, the test statistic (e.g. *F*, *t*, *r*) with confidence intervals, effect sizes, degrees of freedom and *P* value noted<br>*Give P values as exact values whenever suitable.*
☒	☐	For Bayesian analysis, information on the choice of priors and Markov chain Monte Carlo settings
☒	☐	For hierarchical and complex designs, identification of the appropriate level for tests and full reporting of outcomes
☒	☐	Estimates of effect sizes (e.g. Cohen's *d*, Pearson's *r*), indicating how they were calculated

Our web collection on statistics for biologists contains articles on many of the points above.

Software and code

Policy information about availability of computer code

Data collection	Data collection was conducted via the PPMI ida platform https://ida.loni.usc.edu/ and initial assessment and curation done via microsoft excel and R
Data analysis	R (version 3.6.3) packages: caret (version 6.0.90), splitshapestack (version 1.4.8), iml (version 0.10.1), minfi (version 1.42),wateRmelon (version 2.2.0)<br><br>Linux: plink (version 1.90 beta 3.38), PRSice2 (version 2.2.8)<br><br>All codes are available at https://github.com/Rrtk2/PPMI-ML-Cognition-PD

For manuscripts utilizing custom algorithms or software that are central to the research but not yet described in published literature, software must be made available to editors and reviewers. We strongly encourage code deposition in a community repository (e.g. GitHub). See the Nature Portfolio guidelines for submitting code & software for further information.

Data

Policy information about availability of data

All manuscripts must include a data availability statement. This statement should provide the following information, where applicable:

- Accession codes, unique identifiers, or web links for publicly available datasets
- A description of any restrictions on data availability
- For clinical datasets or third party data, please ensure that the statement adheres to our policy

Data used in the preparation of this article were obtained from the Parkinson's Progression Markers Initiative (PPMI) database (www.ppmi-info.org/access-dataspecimens/download-data). For up-to-date information on the study, visit ppmi-info.org.

Human research participants

Policy information about studies involving human research participants and Sex and Gender in Research.

Reporting on sex and gender	In this study we utilize sex, in reference to the biological attribute and refer to it as such in our manuscript. Findings apply to both males and females and we detail methods used to stratify the populations tested to account for both sexes. We provide demographic sex distributions of tested groups within this study in table 1 of the manuscript. Sex measures are operationalized within PPMI based on patient records and were validated for available subjects based on genotype information.
Population characteristics	Relevant population characteristics are outlined in Table 1 for Parkinson's cases retaining normal cognition (n = 127), developing MCI (n = 39) or Dementia (n = 43) present within PPMI.
Recruitment	Analysis was conducted on the PPMI Parkinson's de-novo cohort, acquired through an application gated IDA portal. Details of PPMI recruitment are detailed online (https://www.ppmi-info.org/study-design/research-documents-and-sops)
Ethics oversight	Study design details were included in the original PPMI access application.

Note that full information on the approval of the study protocol must also be provided in the manuscript.

Field-specific reporting

Please select the one below that is the best fit for your research. If you are not sure, read the appropriate sections before making your selection.

☒ Life sciences ☐ Behavioural & social sciences ☐ Ecological, evolutionary & environmental sciences

For a reference copy of the document with all sections, see nature.com/documents/nr-reporting-summary-flat.pdf

Life sciences study design

All studies must disclose on these points even when the disclosure is negative.

Sample size	Of a total 423 available de-novo PD cases, analysis was conducted on cases retaining normal cognition (n = 127), developing MCI (n = 39) or Dementia (n = 43).
Data exclusions	As detailed in Figure 1 of the manuscript, exclusions were based on: impairment at baseline, lack of longitudinal observations, lack of MDS criteria cognitive diagnosis, cases with a reverting longitudinal phenotype. Genetic and epigenetic data point exclusion was conducted as detailed in supplementary methods based on quality control criteria. CSF measures data point exclusion as detailed in methods
Replication	Replication was not viable in this study at current, due to the breadth of variables tested and the cognitive outcome used. This is justified and explained further in our discussion.
Randomization	Random stratified sampling was used for defining training and testing datasets, as detailed in methods
Blinding	Blinding was not possible in this case as group-wise stratification required prior knowledge of outcome groups.

Reporting for specific materials, systems and methods

We require information from authors about some types of materials, experimental systems and methods used in many studies. Here, indicate whether each material, system or method listed is relevant to your study. If you are not sure if a list item applies to your research, read the appropriate section before selecting a response.

Materials & experimental systems

n/a	Involved in the study
☒	☐ Antibodies
☒	☐ Eukaryotic cell lines
☒	☐ Palaeontology and archaeology
☒	☐ Animals and other organisms
☒	☐ Clinical data
☒	☐ Dual use research of concern

Methods

n/a	Involved in the study
☒	☐ ChIP-seq
☒	☐ Flow cytometry
☒	☐ MRI-based neuroimaging
